# Phosphorus—Its Use in Medicine

**Published:** 1877-05

**Authors:** 


### Selections.

---

Phosphorus—Its Use in Medicine.—In a continued article (*Jour. of Materia Medica* of April, 1877) we find the following interesting remarks on the use of phosphorus:

In small doses it would appear to stimulate the stomach; for patients tell us that it produces a sensation of warmth at the epigastrium, and that it increases the appetite. In this way it appears to favor nutrition; it likewise increases the pulse and the temperature. One disagreeable effect is the eructation of phosphureted hydrogen, but this need not occur if it be properly administered. It is chiefly noticed when given in a fluid form.

The general stimulant effects noticed are accompanied by increased cerebral activity and increased muscular power, which implies increased tissue change. We find that it increases the action of the skin and kidneys. Under its influence also, the menstrual flow is often augmented. We are under the impression, too (though we do not find it noticed by writers), that it increases the biliary and intestinal secretions. Certainly, some patients cannot take it on account of its irritating the bowels. Phosphorus is eliminated chiefly by the kidneys. Probably a portion of it may also be eliminated by the alimentary canal, as in poisonous doses this is certainly the case, as previously mentioned. It certainly increases the excretion of phosphates by the urine; and we have observed clinically—a fact not noticed in books—that it removes deposits of urates, whether by improving the digestion or increasing the amount of water excreted, or otherwise, is worth investigating.

Having thus considered its general effects, we are prepared to glance at the various diseases in which phosphorus has been found useful, and the special indications for its employment.

If we were required to classify the properties of phosphorus under the old-fashioned terms, we should say it is a stimulant and tonic, perhaps also analeptic, and in respect to each of these possessed of a general and special action. The term alterative would also have to be resorted to in reference to some of its supposed qualities. We shall, however, consider ourselves free from this kind of classification, though we shall follow it so far as to give some order to our observations.

First of all, then, in certain general chronic diseases attended by debility and wasting, phosphorus has been found beneficial. Here it is that phthisis, tuberculosis, and various cachetic conditions, as well as the consequences of exhausting discharges, prolonged suppuration, hyperlactation, or overwork, may be considered. In such cases the hypophosphites have long been employed. These compounds have, in France, been extensively quacked as specifics for consumption, and the reflex of that quackery has reached our own country. That in properly selected cases they may probably do good seems to be shown by the observations of Drs. Thorowgood and Risdom Bennett, but it is not improbable that the pure metalloid will be found to replace them often with advantage. As a rule, if they disagree with the stomach they do harm, whereas when they appear to promote digestion and increase flesh they should be persevered with. When cod-liver oil seemed to be indicated it has sometimes been made the medium, but the taste of phosphorized oil is so nauseous that patients often refuse to take it; and as it is desirable they should not be disgusted with their oil, it is better for the metalloid to be taken in pills during a course of the oil or its emulsion. In consumption, in struma, in rickets, in mollities ossium, and in other cases affecting the bony system, this treatment seems to have some claim to be considered analeptic as well as tonic.

As a diffusible stimulant, as evinced by its effect on the vascular and muscular systems, it has also been employed in cases of profound adynamia, and some continental physicians think it useful in the typhoid state, and others have resorted to it as a stimulant in fevers and pneumonia. Dr. H. C. Wood, Jr., has seen it act favorably in such cases, but we need further experience in these cases.

In diseases of the skin, Drs. Eames, Percy, and others have attributed to it a value which may, perhaps, entitle it to be considered an alterative. They have tried it in lupus, psoriasis, acne, and furuncular affections. Its analogy to arsenic has been sufficiently obvious to lead Dr. Broadbent into a speculation as to its uses in skin diseases—a good deal more to the purpose than his hypothesis about its value in leukæmia. It has long been supposed that in the exanthemata, when the eruption recedes, phosphorus promotes its reappearance on the skin, and we have already shown that it stimulates perspiration.

On the glandular system its alterative or stimulant effects remain uncertain. Goitre has been known to recede under its influence, and the same may be said of enlarged lymphatic glands, but other remedies are in such cases far more effective, and it is probable that those which have been benefited by phosphorus would have done just as well, or even better, under tonics, iodine, or cod-liver oil. Indeed, in not a few cases the phosphorus has been given in combination with these agents, and to them, very likely, most if not all the effect has been due.

Tavignot in France, and Gioppi in Italy, have employed phosphorus in certain forms of cataract, and others have prescribed it in what they have called amaurosis, and Mr. Jabez Hogg has found it useful in atrophy of the optic nerve.

Diseases of the nervous system are unquestionably those in which phosphorus has the most claim to our attention. Here it claims a *role* which no other drug can pretend to play. Here, doubtless, it acts as a nutrient as well as a tonic. Certainly it appears to be worth a trial in an immense number of cases for which, until recently, there was no resource except rest, fresh air, perhaps sea air, and phosphorized food; in cases of exhaustion of the nervous system, so commonly induced in fashionable life, it has no substitute, and it has favorable influence in organic disease, whether cerebral or spinal; of course, in all such cases it should not be tried in the acute stage, or in stimulating doses, which might be injurious. In chronic white softening of the brain, and in paraplegia following myelitis, it has been prescribed with varying success, and may be given with iron when we have reason to believe that the condition of the nervous centres is anæmic. Dujardin-Beaumetz has tried it in progressive locomotor ataxia, and although he has not seen any cases cured, he believes that some have been decidedly benefited. Bartholomew has used with benefit phosphorized cod-liver oil in paralysis agitans. Delpech has treated a number of cases of paralysis of various forms by phosphorus. The same author reports that it is of especial value in the peculiar cachexia which affects the workers in india-rubber. He thinks that the bisulphide of carbon to which they are exposed acts as a solvent on the phosphorized brain fat, and that given as a medicine phosphorus may supply the loss. Turning to the peripheral nervous system, Dr. Anstie considers it of not much value in neuralgia, but others have met with great success, especially in intercostal and trigeminal. Those who look upon neuralgia as due to exhausted nerve power, would naturally expect that phosphorus might relieve it. Mr. Ashburton Thompson now gives it in large doses, as much as one-twelfth of a grain, though he formerly employed small ones. It should be remembered that doses of one-thirtieth have set up toxic symptoms. In the nervous affections of the aged, accompanied with feebleness of memory, trembling, and cramps, it has been found useful. The wakefulness of aged patients, which is often so troublesome, may often be rapidly relieved by minute doses. Full doses should never be given to old people. In cases of early decay of the mental powers it has been strongly recommended, as well as in cases of break-down from over-work. In impotence it has been empirically prescribed, as well as for various consequences of sexual excess. Acton and others have given it with marked success in the cachexia induced by masturbation. If it be combined with iron, and care exercised in discriminating the cases, the effect in restoring mental vigor is often remarkable. It should be employed with caution, and never when there is any tendency to plethora, cerebral congestion, or hæmorrhage.

The dose of phosphorus, according to the text-books of materia medica, varies from one-fortieth to one-eighth of a grain, but we consider the last much too high to be safe. Mr. A. Thompson prefers large doses, but we do not. In a case of accidental poisoning he gave as much as half a grain every twenty-four hours for six days, and thinks this amount "not excessive." We do, and the numerous cases in which toxic effects have followed less doses support our opinion. We agree with Dr. Kirby that it is safer and more efficacious to give small doses repeatedly than to "surprise the system" with a large dose. We consider one-twentieth of a grain a medium dose, and from one-twentieth to one-fifteenth a full dose. If repeated, it is wise not to exceed a quarter grain the twenty-four hours, and each dose should be taken with a meal.

*Preparations.*—The object being to introduce free phosphorus into the system, we should avoid all preparations which expose the phosphorus to oxidation during administration or after it has been taken. Solutions in oil are rather defective in this sense, and besides being extremely nauseous, they are apt to set up gastric irritation, and give rise to the formation of phosphureted hydrogen. Ethereal and alcoholic solutions possess these disadvantages also, and in consequence of their strength varying considerably with their age, are furthermore dangerous. Mr. Thompson says his tincture may be given in larger doses than other solutions—a proof, to our mind, that some of the phosphorus is precipitated in dilution, and therefore not taken at all. To give an ethereal solution pure, as some have talked of, is a safe method of detaining the phosphorus *somewhere* on its way from the bottle to the stomach. It is said, and rightly, that *solid* phosphorus should not be given; but Macnamara, in his last editions of "Neligan," showed that this observation does not apply to pills carrying the metalloid in solution. For this reason he advised suet as an excipient. The pill of the British Pharmacopœia should be rejected. It has been shown that it usually passes through the bowels unchanged. Those who have devoted themselves to the improvement of pharmacy have directed attention to the preparations of phosphorus. Among these we are glad to find some members of the profession, and it would be well if others were to follow their example. Tilden, of New York, and Dr. Kirby, of London, have both devised processes for the preparation of phosphorus pills, and Dr. Kirby's process is employed by his sons on a very large scale. Both these physicians have succeeded in converting a *solution* of *phosphorus* into a mass of such consistence that it is easily (with proper excipients) made into pills. By Dr. Kirby's process this is done without exposing the phosphorus to oxidation, and it is so well held by the excipients that it does not give rise to any local irritation in the stomach, and is not followed by eructations of phosphureted hydrogen. He has managed to effect this without allowing it to become oxidised. To accomplish this entails, we hear, much care and skill, as well as some danger, throughout the manipulation. Nothing can be easier than to make powdered phosphorus into pills, and we fear that most of the pills in the market are of this sort. Hence they are uncertain, and consequently dangerous. Other specimens are so hard and insoluble that, as in those of the Pharmacopœia, the absorption of the dose is altogether conjectural. We have not been able to meet with Tilden's pills in London, but we have examined some made by Messrs. Kirby, and found them to possess the qualities indicated. Any one can easily test these. Break up in the fingers and roll together half a dozen of these pills in a dark room, and they will be seen to be luminous, and the phosphorescence may be observed to be uniform through-the mass. We have watched this for above an hour. Every time the mass is cut or broken, or indented with the nail—in fact, whenever a new surface is exposed, fresh gleams appear, and this phosphorescence is not in points, but uniformly diffused. The total absence of anything like particles of phosphorus is very satisfactory, and we believe that the method of preparing them must be as represented, by dissolving the phosphorus first, and afterwards adding the excipients, so as to preserve throughout the process the advantages of solution. We could suggest formulæ for this, but the trouble and danger involved in the manipulation would prevent any one preparing them on a small scale. The leading London pharmaceutists dispense Kirby's pills, and these are always obtainable. Few care to incur the responsibility of attempting to make their own. These pills have the advantage, too, of dissolving slowly, do not irritate the stomach so much, and are believed by many, with some degree of probability, to be only completely dissolved in the duodenum, so that the free phosphorus, mingled with the aliments, finds its way into the blood.

Phosphorized oil, in capsules, has also been tried, but with little satisfaction. Apart from the uncertainty of the amount they contain, and the difficulty of making them, the capsule envelope is itself liable to great variation, and seldom obtainable of good quality. In France Dr. Clertan has indeed brought the manufacture of capsules in the form of his *perles* to great perfection for ether, chloroform, turpentine, and other drugs. If disposed to try capsules, we should certainly prefer his manufacture, but we do not think this form equal to that of pills.

Before closing, we are bound to advert to some published formulæ worthy to be spoken of as curiosities of pharmacy, except for the fact that some are dangerous. They pass from one book to another unchallenged and without comment, and authors whom we should have thought better informed on the pharmacy of phosphorus, have quoted them, though with what purpose, as they are practically useless, it is difficult to divine. Why perpetuate obsolete prescriptions which are likely to do harm? We have met with some of these in which directions impossible to follow are given. Thus as late as 1873 we actually find this credited to *Burgess*, in a work on skin diseases:

| | |
|---|---|
| ℞.—Phosphorus, . . . . | g. iij. to xx. |
| Almond Oil, . . . . . . | gtt. x. to lx. |
| Powdered Acacia, . . . | q s. |

Make twelve pills.

Dose, one twice a day. Use in lupus and syphilitic tubercular disease.

How many persons have been poisoned by this it may be difficult to say. Perhaps the difficulty of dispensing the prescription may have saved some lives, but we are astonished beyond measure to find a careful teacher admitting into his work a formula which is too defective to attempt to prepare. If by some means ten or twenty grains of it was got into twelve pills, most assuredly the person who took them would be *poisoned.* Twenty grains of phosphorus in twelve pills!!

A very similar formula was for many years a standing dish in Hooper's "Physician's Vade Mecum." In the last edition we are glad to see it omitted. With such formulæ floating about, we cannot be surprised that phosphorus acquired the reputation of being a violent irritant poison; but during the last few years it has been given right and left, and is taken just like any other tonic, and we hear nothing of poisoning, except in a few solitary cases, in which it has been clearly proved that its dose was excessive or its preparation defective. *The Doctor.*

